# Few-Shot Text Classification with Global–Local Feature Information

**DOI:** 10.3390/s22124420

**Published:** 2022-06-11

**Authors:** Depei Wang, Zhuowei Wang, Lianglun Cheng, Weiwen Zhang

**Affiliations:** 1 School of Automation, Guangdong University of Technology, Guangzhou 510006, China; 1111904019@mail2.gdut.edu.cn; 2 School of Computer Science and Technology, Guangdong University of Technology, Guangzhou 510006, China; wangzhuowei0710@163.com (Z.W.); llcheng@gdut.edu.cn (L.C.)

**Keywords:** text classification, few-shot learning, news categorization, feature selection

## Abstract

Meta-learning frameworks have been proposed to generalize machine learning models for domain adaptation without sufficient label data in computer vision. However, text classification with meta-learning is less investigated. In this paper, we propose SumFS to find global top-ranked sentences by extractive summary and improve the local vocabulary category features. The SumFS consists of three modules: (1) an unsupervised text summarizer that removes redundant information; (2) a weighting generator that associates feature words with attention scores to weight the lexical representations of words; (3) a regular meta-learning framework that trains with limited labeled data using a ridge regression classifier. In addition, a marine news dataset was established with limited label data. The performance of the algorithm was tested on THUCnews, Fudan, and marine news datasets. Experiments show that the SumFS can maintain or even improve accuracy while reducing input features. Moreover, the training time of each epoch is reduced by more than 50%.

## 1. Introduction

Text classification is a universally acclaimed task in information extraction. This task is defined as predicting a label for a given text from a label pool. Recently, deep learning has generated great success, thanks to optimization algorithms, large datasets, and model architectures. Based on word vectors, semantic information can be obtained by characterizing each word as a dense vector [1]. By embedding such information, diverse deep learning architectures for text categorization have been proposed. These architectures range from superficial stacking layers, such as a recurrent neural network (RNN) and convolutional neural network (CNN), to deeper ones. Additionally, more sophisticated structures have been proposed, such as transformer or graph neural network (GNN) models. However, these models suffer from data deficiency and heavy calculations, which are labor-intensive and time-consuming.

Few-shot learning has been said to solve the problem of data deficiency by identifying new classes from a few labeled samples. In recent years, few-shot learning has been generously applied to computer vision tasks and is emerging as a promising solution to the low-resource regime [2]. Some researches have achieved better results on text classification tasks [3,4] by focusing on the local-to-global paradigm, which minimizes the distance between the support and query distributions to obtain strong performance over the entire dataset. Moreover, feature selection is a way to reduce time consumption. It is used to enhance classification accuracy by eliminating irrelevant data [5,6].

In practice, text is a collection of semantic information. The metric-based models rely on the spacial distribution of semantic feature representation in the embedding space. Previous few-shot learning works still suffer from key feature and combined features extraction. Additionally, Few-shot learning methods are not extensively investigated in natural language processing (NLP), motivating us to explore a meta-learning framework for NLP.

In this paper, we improve the performance of low-labeled text classification with fewer inputs. Specifically, few-shot learning based on the extractive summarization approach (SumFS) is proposed for low resource text classification tasks. The method adopts a global-to-local strategy. It adjusts the global distribution of the dataset and optimizes data spacing within each learning task. First, the purpose of an extractive summary is to find the global top-ranked sentences, characterizing category information, with unsupervised methods used on the raw text. Second, the weight of each word in the text is calculated, and the local sentence category features are improved. Third, meta-learning attempts to learn model parameters quickly to improve the predictive performance of the test sets by using training examples for each class. Fourth, the ridge regression classifier can prevent overfitting and achieve fast adaptation given limited data. In other words, our approach simplifies the amount of input text and applies fewer weighted features to texts with strongly correlated categories. To summarize, our contributions are three-fold:

1 The SumFS algorithm is proposed to implement text pre-processing and small sample classification tasks in a pipeline processing manner;
2 We propose a global-to-local strategy that utilizes the maximum dataset distribution distance to minimize the category distances within each learning task;
3 Extensive experiments on the datasets were conducted, along with detailed comparative analysis, which demonstrates that our proposed method is superior to its alternatives.

The remainder of this paper is organized as follows. Section 2 introduces related work. Section 3 presents the proposed method. Section 4 describes the experiment’s performances. Section 5 summarizes our research and gives recommendations for future work.

## 2. Related Works

### 2.1. Deep Learning for Text Classification

Deep neural network models have played an essential role in text classification in the past few decades. RNNs and CNNs are the initial popular network frameworks. With the deepening of research, several joint models have emerged. Lee and Dernoncourt [7] combined RNN and CNN methods for solving sequential short text classification problems. Wang et al. [8] combined RNN and Capsule to achieve state-of-art sentiment classification. The excellent performance of RNN(s) and CNN(s) in terms of their classification results have also triggered broader exploration of the interpretability of the classification results. Moreover, the attention mechanism is introduced to interpret the hidden layer data processing results, assisting the understanding of what the model has learned and supporting the visualization of the output results. Lai et al. [9] used a multi-head attention model and attention mechanism to enhance sentence representation in a sequence information extraction task. In addition, the series of models based on Bert [10] have achieved significant results in global semantic information acquisition and obtained better representation capabilities with pre-training methods. Yang et al. [11] proposed XLNet to overcome the ignorance of location information in Bert and incorporated outperformers in question answering (QA), sentiment analysis (SC), and document ranking (DR). Furthermore, GNN converts text analysis from a conventional structure to a graph structure, and transforms the task of text classification into one of graph-node classification. Differently from general semantic information, GNN captures syntactic structure information. Zhang et al. [12] proposed TextING to overcome regular GNN’s weakness in the correlation between words across lines and new word induction learning ability.

Overall, the depth and width of deep learning models are increasing: the success of these deep learning models is based on substantial task-related datasets, and this gives rise to many problems, such as over-reliance on a dataset, time cost, and their cumbersome nature. Therefore, the input dimension is reduced to accelerate model training in few-shot learning.

### 2.2. Meta-Learning for Text Classification

The research enthusiasm for few-shot learning continues to rise, and the applications are gradually expanding in number. Meta-learning is a popular technique used in few-shot learning, which learns through multiple assistance tasks. The latest meta-learning explorations are typically divided into three separate categories, including learning fitted weight initialization to adapt quickly to new tasks [13], learning transferable optimization algorithms [14], and learning parameters to initialize the weights of another network [15]. MAML is one of the most well-known meta-learning frameworks [13], which has achieved advanced results in many experimental settings because of its effectiveness. Some studies focus on accelerating the MAML model, simplifying the computational complexity [16], and reducing the dimensionality [17]. Noteworthy advances have been investigated in terms of machine translation [18], text classification, and relationship classification [19].

In the context of few-shot text classification, Pan et al. [14] proposed an improved layered pool and a text classification strategy based on pre-trained word embedding. Bao et al. [3] trained a meta-learning framework to map feature codes into attention scores for measuring the vocabulary representations of words, and adopted the ridge regression classifier to predict the results after seeing only a few training examples. Pang et al. [20] used a bi-directional attention mechanism to encode the classification features. Despite recent progress, the generalization abilities of existing models are limited, and meta-learning application scenarios are in urgent need of improvement.

### 2.3. Feature Selection for Text Classification

Text classification tasks usually represent the text as a high-dimensional vector, and the high dimensionality brings a series of issues, such as the curse of dimensionality and overfitting. Deng et al. [21] gave a comprehensive review on feature selection techniques for text classification. Mirończuk and Protasiewicz [22] divided the dimensionality reduction strategy into three parts: feature selection, feature mapping, and instance selection. The first two methods aim to abate the feature space dimension, and the third method aims to lower the number of training examples. Awan et al. [23] proposed TOP-Rank to extract the most relevant key phrases from text and then perform classification. Pintas et al. [5] classified term-frequency-inverse-document-frequency (TF-IDF) as an unsupervised technique in the survey of feature selection. Tang et al. [24] used matrix factorization techniques to reduce features when dealing with short social media texts. Khurana and Verma [25] introduced a new algorithm to solve high-dimensionality, named modified biogeography-based optimization, which uses a feature weighting algorithm to modify the ranking of variables. Rashid et al. [26] proposed new k-means topic modeling to capture better semantic topics.

In the study of unsupervised feature selection methods, Behera and Kumaravelan [27] used both TF-IDF and CNN-based feature extraction methods for both text classification and feature selection on a fuzzy rough set. Watanabe et al. [28] studied numerous pre-processing and feature extraction methods, such as tokenization, stop-word removal, word lemma, decision tree TF-IDF, and support vector machines as classification algorithms in the application of systemic literature review tasks. Tang et al. [29] proposed several alternative methods inspired by the IDF part of TF-IDF (defined as logarithmic transformation) and applied various chosen methods to generate unsupervised item weighting schemes to compensate for the shortcomings faced by TF-IDF. Liu et al. [30] proposed an unsupervised text representation method that included a WWE model based on TF-IWF and CBOW. They used TF-IWF to weight the words in the vector for short texts. Our work combines feature selection and instance selection methods, which benefit from the unsupervised abstract technology in text classification tasks [31]. For sentence-level features, we use the TextRank method to obtain top-ranked sentences as input text and the IDF-IWF method to determine word-level features, with different weights to enhance semantic information.

## 3. The Proposed Method

**Figure 1:**
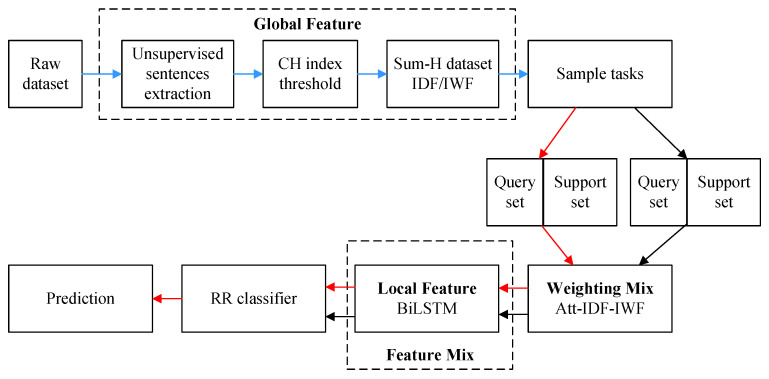
The SumFS framework. The blue arrow denotes the global feature processing process, the black arrow denotes the training process, and the red arrow denotes the testing process.

Our proposed framework, SumFS, is shown in Figure 1. It consists of global–local feature extraction and a meta-learning framework. The global feature extraction module aims to decrease text dimensionality and enhance word features. The unsupervised text summarization method is used to select top-ranked sentences which contain more semantic information and better fit the text subject. Then, the Calinski–Harabaz (CH) index is selected as the threshold to determine how many sentences are extracted. IDF/IWF methods are adapted to realize the mining of word-level features. The local feature measures the various contributions of a word to a class via attention mechanism. The feature mix module adds feature weights to word vectors derived from BiLSTM. Finally, the Sum-H dataset was used to train a meta-learning model with a ridge regression classifier.

The pseudo-code of the SumFS model is presented in Algorithm 1. The following is a summary of the process of the SumFS model for few-shot learning. First, we leverage the TextRank method for unsupervised text summarization. Meanwhile, the CH index determines the number of sentences extracted from each text. Then, the training and validation data can be used to train the SumFS model. Weighting embedding is achieved using word weight feature selection methods. After the training process, the classifier is trained under the loss function. Finally, the unseen test data were employed to evaluate the model.

**Algorithm 1:** Pseudo-code for SumFS.

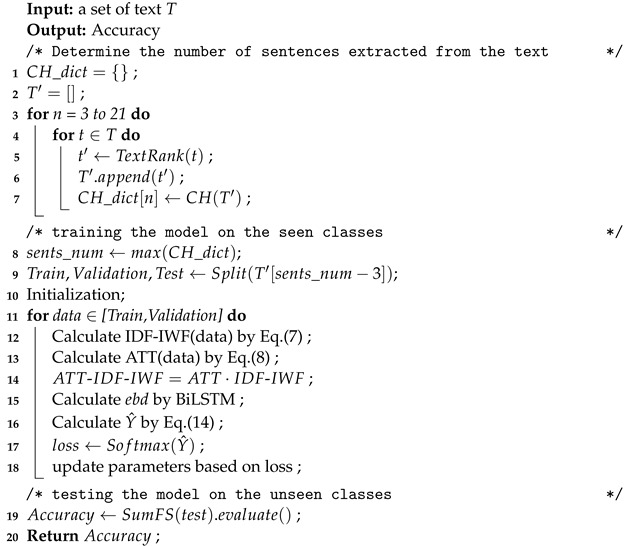



### 3.1. Sentence Extraction

Text summarization is the process of producing a short version of a document by preserving the essential information of the document as much as possible. Extractive summarization is a direct way to capture the main information of a text. Mihalcea and Tarau [32] proposed the TextRank algorithm, and this method has also been used for unsupervised, extractive text summarization [31]. The traditional TextRank algorithm characterizes the interactions between nodes in a graph. The first step of key sentence extraction is to construct an interactive graph model of sentence units. Then, it uses the PageRank algorithm [33] to calculate the weight of each sentence (node) and ranks nodes according to the weights assigned thereto. The input text is defined as a directed graph G=(V,E) with $V=S_{1},S_{i},\cdots ,S_{j}$ denoting the set of sentences and *E* denoting the set of edges between sentences. After constructing the graph, the key sentences are extracted as follows. First, the TextRank score of each node in the graph is calculated by PageRank. TextRank score of node $s_{i}$ is defined by Equation (1):

(1) $$TR\left(s_{i}\right)=\left(1-d\right)+d\sum_{j\in \mathrm{In}\left(s_{i}\right)}\frac{\mathrm{TR}\left(s_{j}\right)}{\left|Out\left(s_{j}\right)\right|}$$

where $TR_{s_{j}}$ represents the weight of the sentence $s_{i}$, $In(s_{i})$ is the set of nodes pointing to $s_{i}$, $Out(s_{j})$ denotes the set of nodes that point from the $s_{j}$, and *d* is a damping factor, which is between 0 and 1. Then, the TextRank value is iteratively calculated. The TextRank formula is shown in Equation (2):

(2) $$TR\left(s_{i}\right)=\left(1-d\right)+d\sum_{V_{j}\in \mathrm{In}\left(s_{i}\right)}\frac{\omega_{ji}}{\sum_{V_{k}\in \mathrm{Out}\left(s_{j}\right)}\omega_{jk}}\mathrm{TR}\left(s_{j}\right)$$

where $\omega_{ji}=cos\left(s_{i},s_{j}\right)$ represents the edge weight between node $s_{i}$ and node $s_{j}$. Equation (2) shows that the weight of the node is related to its first-order associated node.

After pre-processing, the order of sentences in a single text is determined. The number of sentences extracted to reduce initial input text affects the degree of dispersion of elements within the category and the distance between categories, ultimately affecting the classification accuracy. Then CH [34] was chosen as an index ( https://scikit-learn.org/stable/modules/clustering.html#clustering-evaluation (accessed on 8 April 2022)), which is the ratio of the sum of between-cluster dispersion and of within-cluster dispersion for all clusters, to calculate the clustering performance under various sentence numbers. At last, *k* sentences with the highest CH index are selected.

### 3.2. Weight Generator

The goal of feature selection is to highlight keywords that contribute information to the classifier. The general weight is defined as TF-IDF [35], which is a classic unsupervised item weighting method. The term weight is mainly composed of the local factor (TF) in the text and the global factor (IDF) in the dataset. Similarly, we extract global features from whole datasets, which can enhance the connections between categories, and local features, which represent the relative contributions of word and category. The inverse document frequency (IDF) measure is used as a global weighting factor to phase the document representation ability of a word *w*. The IDF weight of the item can be expressed as Equation (3):

(3) $$\mathrm{IDF}\left(w\right)=\log\left(\frac{Num}{n_{t}\left(w\right)+1}\right)$$

where $n_{t}\left(w\right)$ represents the number of documents that contain word *w*, and Num is the total number of documents in the corpus.

For another feature extracted from the full datasets, IWF (inverse word frequency), was selected to capture dynamic features. First, WF characterizes the frequency of word *w* at the exact step time *t*, as given by Equation (4):

(4) $$wf_{t}\left(w\right)=wf_{t-1}\left(w\right)+wf_{s_{t}}\left(w\right)$$

where $s_{t}$ represents new data at time *t*. $wf_{s_{t}}\left(w\right)$ denotes the number of occurrences of words *w* in the newly added text dataset, and $wf_{t-1}\left(w\right)$ represents the number of occurrence of word *w* before time *t*. Second, IWF is denoted by Equation (5):

(5) $$IWF_{t}\left(w\right)=\log\frac{W_{t}+1}{wf_{t}\left(w\right)+0.5}$$

where $W_{t}$ is the total number of occurrences of all words in the corpus *d* before time *t* and calculated in Equation (6). The function Freq(·) is used to calculate the word frequency.

(6) $$W_{t}=\sum_{t^{\prime }\leq t}\sum_{w^{\prime }\in d}Freq\left(w^{\prime },d\right)$$

Then, the use of an IDF-IWF method was proposed to improve the significance of global feature acquisition for the weighting of word *w* on all datasets, with the IDF-IWF value calculated by Equation (7):

(7) $$IDF*IWF=IDF\left(w\right)*IWF_{t}\left(w\right)$$

It is natural to ask whether other weighting methods can be integrated as efficiently as IDF-IWF within a meta-learning framework. Another local weighting strategy is the attention mechanism. It allows the model to have more direct dependencies between states at different times. Note that the model is used to weigh contextual features and assign different weights to each word. All hidden states are weighted together in the weight assignment strategy:

(8) $$c_{t}=\sum_{j=1}^{T}\alpha_{tj}h_{j}$$

where *T* is each whole time-step contained in the input sequence and $\alpha_{tj}$ is the weight computed for each state $h_{j}$ at exactly *t*. In the Bi-LSTM model (Section 3.3), its structure overcomes the long-term dependency bottleneck of RNN, so current $s_{t}$ depends on $s_{t-1}$, $c_{t}$, and the model output at t−1. The weights $\alpha_{tj}$ are then calculated using Equation (9):

(9) $$e_{i,j}=a\left(s_{t-1},h_{j}\right),\alpha_{ij}=\frac{\exp\left(e_{tj}\right)}{\sum_{k=1}^{T}\exp\left(e_{tk}\right)}$$

where a(·) is a learnable function. It can be considered to calculate the state weightness value for $h_{j}$ given the value of $h_{j}$ and the previous state $s_{t-1}$. Equation (9) allows the next state sequence *s* to directly access the entire state sequence *h*. To this end, we have obtained three combined features denoted as IDF-IWF-ATT.

### 3.3. Sentence Embedding

RNNs excel at handling sequence data and preserving contextual information and have allowed rapid progress in text representation. The bidirectional long short-term memory network (Bi-LSTM) [36] is a further development of the regular RNN to increase the input information diversity, solving long-term dependency problems. Compared with the one-way LSTM network, which consists of repeating modules (cell), BiLSTM adopts a forward and backward sequence training strategy to capture context information. For the selection about input information, it sets a gate function unit to control the cell state: the unit is made up of an input gate *i*, output gate *o*, and forget gate *f*. After the choosing of gate units, each hidden state $h_{t}$ is calculated by Equation (10):

(10) $$\left\{\begin{array}{c} f_{t}=\sigma \left(W_{f}\cdot \left[h_{t-1},x_{t}\right]+b_{f}\right)\\ i_{r}=\sigma \left(W_{i}\cdot \left[h_{t-1},x_{t}\right]+b_{i}\right)\\ {\tilde{C}}_{t}=\tanh\left(W_{c}\cdot \left[h_{t-1},x_{t}\right]+b_{C}\right)\\ C_{t}=f_{t}^{*}C_{t-1}+i_{t}^{*}{\tilde{C}}_{t}\\ o_{t}=\sigma \left(W_{o}\left[h_{t-1},x_{t}\right]+b_{o}\right)\\ h_{t}=o_{t}^{*}\tanh\left(C_{t}\right) \end{array}\right.$$

where $W_{f}$, $W_{i}$, and $W_{o}$, respectively, represent the connecting weights between cells and gates. The inputs to each cell unit are $h_{t-1}$ and $x_{t}$. Three control gates are $\left(f_{t},i_{t},o_{t}\right)$; $b_{f},b_{i},b_{o},b_{C}$ are bias terms. The ${\tilde{C}}_{t}$ and $C_{t}$ are the vectorized representations of the cell states. They are critical in the LSTM cell chain. The stable state transmission allows BiLSTM to process them in a bi-direction manner. Next, we pick up the hidden layer output $H=(\vec{h},\overset{\leftarrow }{h})$ in two directions. $\vec{h_{t}}\;\mathrm{and}\;\overset{\leftarrow }{h_{t}}$, respectively, represent the hidden layer outputs processed by the positive and negative LSTM at time *t*. Thereafter, the sigmoid activate function θ(·) and hyperbolic tangent function tanh(·) are multiplied to form the output result. Finally, the output of the final hidden layer $h_{t}$ is given by Equation (11):

(11) $$h_{t}=\vec{h_{t}}⨁\overset{\leftarrow }{h_{t}},h_{t}\in R^{2C}$$

where ⨁ concatenates the bidirectional output, and *C* represents a unidirectional LSTM network size.

### 3.4. Classifier

With multiple tasks, meta learning can be typically regarded as an *N*-way *K*-shot classification problem. In a specific single task, *N* represents the number of specific categories, and *K* represents the number of labeled samples in each category. After the aggregation of the total sample data included in entire tasks, it can be represented by $D=\left\{\left(x_{1},y_{1}\right),\ldots ,\left(x_{N},y_{N}\right)\right\}$, where *N* is the number of classes in *D*. In particular, an explicit task *T*, $V=\left\{y_{i}|i=1,\ldots ,N\right\}$ denotes the class labels set. The input data of meta learning model is randomly extracted from *D* and divided into a support set and query set: specifically, in task *T*, the support set is pointed to $S=\left\{(x_{i},y_{i})|i=1,\ldots ,m\right\}$, where m=N×K (*N*-way, *K*-shot); the query set is $Q=\left\{(x_{j},y_{j})|j=1,\ldots ,n\right\}$, where *n* is a sample data size selected for meta-testing [37].

Meta-learning algorithms try to use meta-training tasks to obtain a learning strategy that can be extended to completed tasks $T^{\prime }∼P\left(T\right)$. After feature selection and use of an embedding layer, the weighted word vector representation is attained. Then ridge regression [3,38] is used as a classifier and trained on the support set, and the regularized squared loss is minimized by Equation (12):

(12) $$L_{i}\left(M\right):={∥SM-Y_{S}∥}_{F}^{2}+\lambda {∥M∥}_{F}^{2}$$

where ${∥\cdot ∥}_{F}$ is Forbenius norm, and λ>0 is a positive hyper-parameter. *M* is given by Equation (13):

(13) $$M=S^{T}{\left(SS^{T}+\lambda I\right)}^{-1}Y_{S}$$

where *I* is the identity matrix.

Although Equation (9) is used to ascertain a good classification effect, the result does not directly output the category label. Thus, it needs to be corrected to apply to the classification of the evaluation test set. The calibration step is given by Equation (14):

(14) $${\hat{Y}}_{Q}=aQM+b$$

where *a* and *b* are the learning parameters. Finally, during the meta-training, the cross-entropy loss between ${\hat{P}}_{Q}=\mathrm{softmax}\left({\hat{Y}}_{Q}\right)$ and the query set labels is calculated. The local attention feature is also trained in a supervised manner.

## 4. Experiments and Results

This section discusses the preparation of the experiments, the setting up of the experimental environment, and the presentation of the experimental results. First, the experimental setup is described in Section 4.1; then, the distribution of original text and unsupervised summary extraction are compared in Section 4.2. The results of the two weighting strategies are compared in Section 4.3. Finally, the classification accuracy and time consumption with different text inputs are compared in Section 4.4.

### 4.1. Experimental Setup

The following experiments were conducted using three datasets: THUCNews, Fudan News, and our Marine news dataset. THUCNews (http://thuctc.thunlp.org (accessed on 8 April 2022)) is a Chinese text classification dataset published by Tsinghua University’s Natural Language Process and Computational Social Science Laboratory. It comprises historical data from Sina News from 2005 to 2011. The entire dataset contains 740,000 news texts, divided into 14 categories. The Fudan news (http://www.nlpir.org/wordpress (accessed on 8 April 2022)) was released by the NLP group at the International Database Center, Fudan University’s Department of Computer Information and Technology. The dataset contains 20 categories, divided according to a 1:1 ratio. The training data contain 9833 documents, and the testing set contains 9804 documents. Marine news, created by the authors, consists of 11 categories of online news texts. The category number in Table 1, corresponds to the order of the categories in Table 2. Regular matching was used to count the average number of sentences contained in each article, which is shown in Table 2.

We expected to randomly select 100 samples in each category, but there were not enough data in some specific categories. For example, there are only 52 samples in the art category of Fudan news, and the same problem also exists in the Marine news dataset. Each dataset was divided into three parts. The training set was applied to train the model, and the validation set was used to terminate the training process early. Additionally, the model’s testing performances were only taken from unseen categories. Table 1 lists the categories used for training, validation, and testing. Additionally, the division was random for each dataset. Experiments were all conducted under this division of data.

We discuss the results in this paper by adopting the accuracy index. Accuracy is a metric for evaluating the fitness of the model’s classifier. The accuracy is defined in Equation (15):

(15) $$ACC=\frac{\mathrm{Number}\;\mathrm{of}\;\mathrm{correct}\;\mathrm{predictions}\;}{\mathrm{Total}\;\mathrm{number}\;\mathrm{of}\;\mathrm{predictions}}\times 100\%$$

Several parameters need to be determined in our experiments in the meta-learning stage. For simple notation, Sum-H represents the number of sentences H extracted from each document in the unsupervised extraction algorithm. Additionally, raw means the original text. In addition, N-way K-shot is simply referred to as N-K. The learning rate is 0.01, the dropout value is 0.1, and the loss function is RMSprop. There were 1000 training epochs, and the early stopping patience was 20. The training, validation, and testing episodes were 100, 100, and 1000, respectively. In addition, the experiments were conducted on a server that was equipped with an Intel CPU E-5-2630 v4 2.20GHz chip-set and an NVIDIA Tesla K80 GPU graphics card. The operating system was CentOS Linux (release 7.9.2009 Core). The code was written in Python programming language.

### 4.2. Clustering Experiment Results

**Figure 2:**
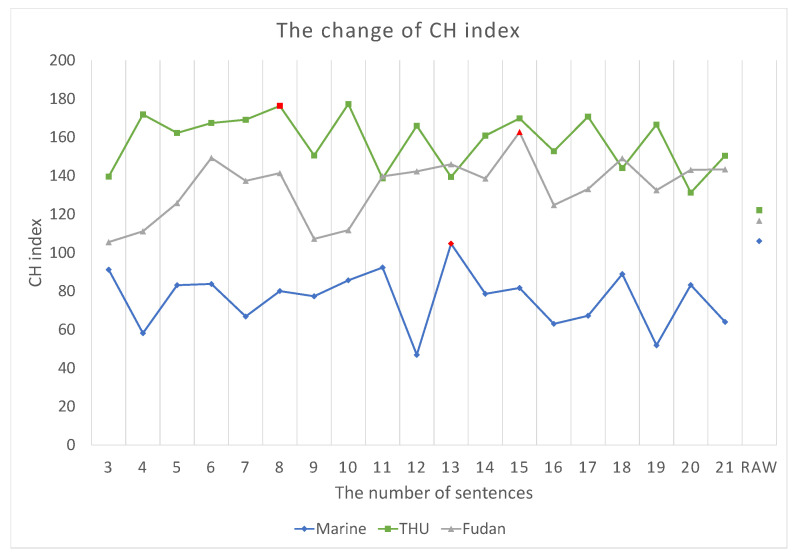
CH index under several Sum-H. The number of sentences corresponding to the red points was chosen.

Different numbers of top sentences were selected to calculate the influences of sentences on the CH index value and compute the raw text CH index value. Figure 2 shows the CH index under different values of Sum-H. The abscissa indicates the number of sentences in a single document, and the ordinate indicates the CH index value. The highest CH index of the Fudan news dataset is 162.69, for sum-15. In addition, we found two relatively close, high CH index values on the THUCNews dataset, which are 176.34 for sum-8 and 177.33 for sum-10. The CH index values of raw are near to sum-13. They are 106 and 104.67, respectively. Moreover, to acquire the minimum input data requirement, sum-8 on the THUCNews dataset and sum-13 on the marine dataset were chosen. In addition, the CH index rates of the Fudan dataset and THUCnews dataset are higher than that on raw text only.

**Figure 3:**
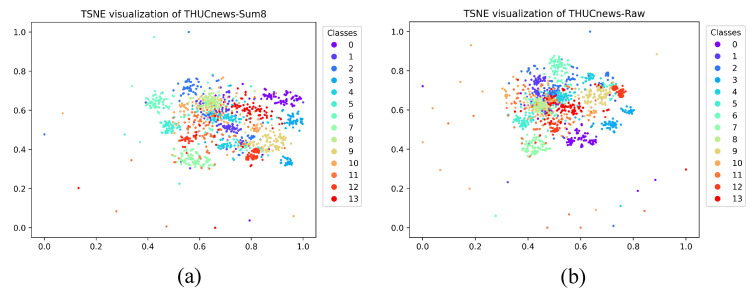

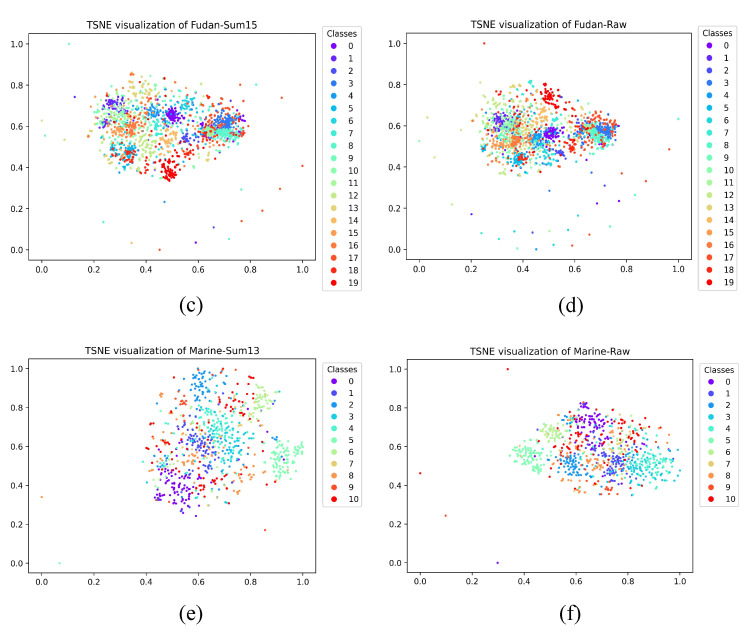
TSNE visualization of the input representations for Sum-H and raw data of three datasets, which are THUCnews, Fudan news and Marine news. Their Sum-H’s distributions are shown in (**a**), (**c**) and (**e**), respectively, and their distributions of raw data are shown in (**b**), (**d**) and (**f**), respectively. Each color/marker pair corresponds to a specific label. The class numbers are listed in Table 1.

Then, the highest CH index value was plotted to observe their clustering results compared with the raw text. Consequently, the category information was not reduced after the dimension reduction operation, but it provided a benefit for category aggregation. An unsupervised extractive text summary was used to obtain high-ranked sentences as model input text. For Figure 3, the t-SNE method was used to illustrate the features of the summary text and the original text clustering more clearly. Compared with the original text, the clustering effect of the text data after the summary is more discrete and increased the distance between categories to a certain extent. The degree of aggregation between each category of abstracted text was also significantly better than that of the original text. In addition, the CH index value of the text after the summary was higher than that in the original text. The number of discrete edge nodes was reduced, which is a significant factor driving the increase in the CH value.

### 4.3. Classification Experiment Results

In this section, we compare different feature weighting strategies on the Sum-H. Furthermore, the classification performance of the proposed model is compared with those of the baseline models, demonstrating its superiority.

Four popular meta-learning training frameworks were selected to perform ground experiment analysis:

1 MAML [13] is compatible with any model trained using gradient descent for different learning problems https://github.com/dragen1860/MAML-Pytorch (accessed on 8 April 2022).
2 Proto [39] performs classification by computing the distance to the prototype representation of each class based on metric space (https://github.com/jakesnell/prototypical-networks?utmsource (accessed on 8 April 2022).
3 R2D2 [3] aims to improve classification performance by learning high-quality attention from the source pool distribution features (https://github.com/YujiaBao/Distributional-Signatures (accessed on 8 April 2022)).
4 MLADA [4] is committed to improving the model’s adaptability. The text representations are obtained by introducing an adversarial domain adaptation network. (https://github.com/hccngu/MLADA (accessed on 8 April 2022)).

The classification accuracy of each model is compared in Table 3. These results were verified when the dataset contained only 100 samples in each category. The MAML model adopts word embedding without weighting information as the input and a multi-layer perceptron as the classifier. The Proto model has the same structure as the prototypical network model, and input embedding does not include weighting information. The R2D2 and MLADA models directly utilize the open-source code. According to the classification results, the MAML model was not successfully trained on all datasets—note the case of a small amount of sample data. The Proto model performed well in the one-shot, but it still feel far short of the results of our method. Although there were no better results with the MLADA model, its performance was entirely satisfactory. Finally, R2D2 and our model’s results are similar, but our results are slightly superior. The key reason is that both methods use weighting strategies and linear classifiers (ridge regression classifiers), which can rapidly improve the model adaptability.

**Figure 4:**
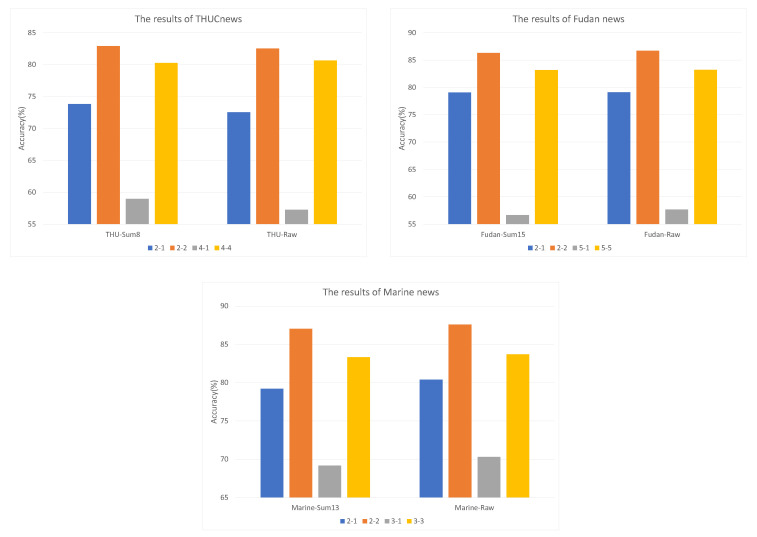
Comparison of IDF-IWF-ATT results with different numbers of sentences.

In addition, multiple comparative experiments were designed to evaluate the proposed model; the classification accuraciesof Sum-H and raw text are compared under IDF-IWF-ATT combined features. The specific classification accuracies are shown in Figure 4. The overall classification results are the same, except for the gray histogram where the changes are more remarkable: the raw text results are better than the results of Sum-H on the Fudan and Marine datasets. On the THUCNews dataset, Sum-8 was 1.31% more accurate than raw text in 2-way 1-shot, and reached 1.7% higher in 4-way, 1-shot. According to 5-way, 1-shot results, Fudan news raw text exceeds Sum-15 by 0.98%. The largest differences in the Marine news dataset were 1.19% and 1.14% between Sum-13 and the raw text. In brief, the unsupervised sentence extraction method maintains text classification accuracy by extracting critical sentences from the raw text.

**Figure 5:**
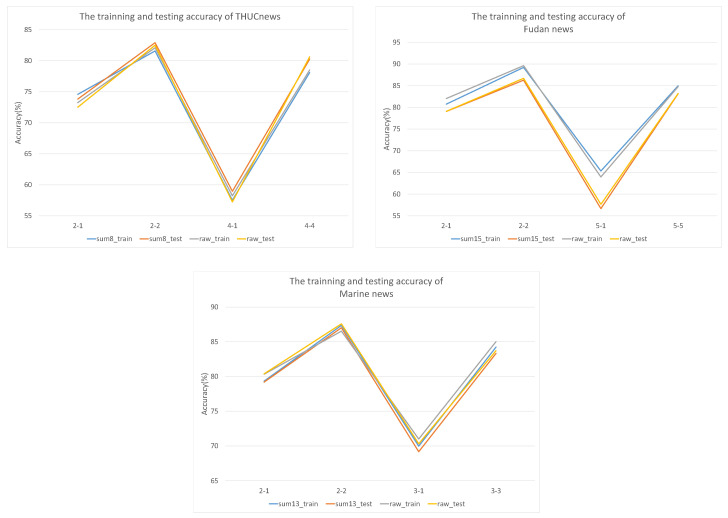
The accuracy trends of training and testing using the ATT-IDF-IWF weighted strategy. According to the results, Sum-H preserves the original text information well.

We also consider whether the trained model can perform stably on the test dataset. According to the laboratory data shown in Figure 5, sum-H and raw text follow similar trends. In addition, the fit was good on both Marine news and THUCnews datasets; and the training results are significantly better than the test results on the Fudan news dataset. Specifically, the test results are 0.43% more accurate than the training results in 4-way, 4-shot; and the maximum difference between training and testing was 8.66% in 5-way, 1-shot. The results show that the text, after being summarized, maintained the same results as the source text, whether it was selected from the training or test data.

We compare five weighting strategies, including TF-IDF, IDF-IWF, IDF-ATT, IWF-ATT, and IDF-IWF-ATT. Table 4 summarizes the results of the classification accuracy on Sum-H. There is no big difference among classification accuracies in the horizontal comparison. Additionally, the top two accuracy gaps are within 0.5. When increasing the K value in multi-classification tasks, the results improved significantly. The classification results on all datasets exceeded 80% when N = K. Furthermore, the optimal classification results are concentrated under one strategy in the Fudan-sum15 dataset. The text sentence length is a pivotal point in explaining this clustering effect. The average text lengths of the three datasets are 151 (THUCnews), 390 (Fudan news), and 240 (Marine news).

**Figure 6:**
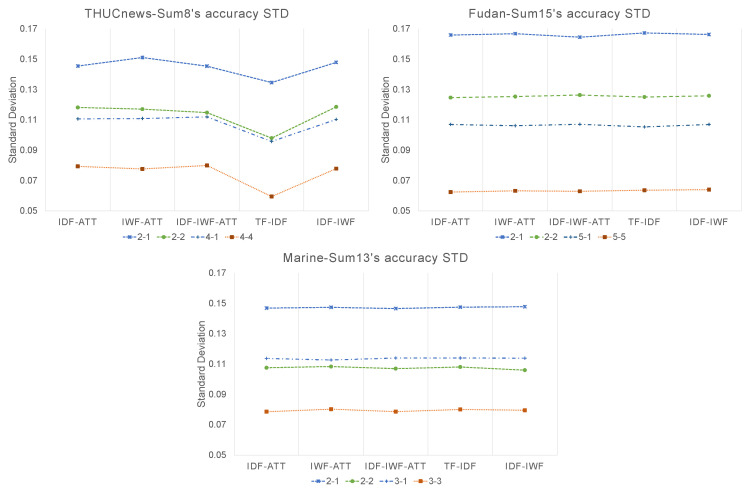
Comparison of the standard deviations during the testing steps.

We also collected the standard deviation (STD) during the testing steps. The results are shown in Figure 6. The STD values are flat for the Marine and Fudan data. All the STDs are lower than 0.17. The STD decreased as the sample size increased. However, THUCnews resulted in the lowest STD value when using TF-IDF. This was probably due to the fact that the weighting method is simple. Another notable fact is that the 2-way 2-shot’s STD is lower than the 3-way 1-shot’s. We infer that this is because the ways’ numbers are similar.

**Figure 7:**
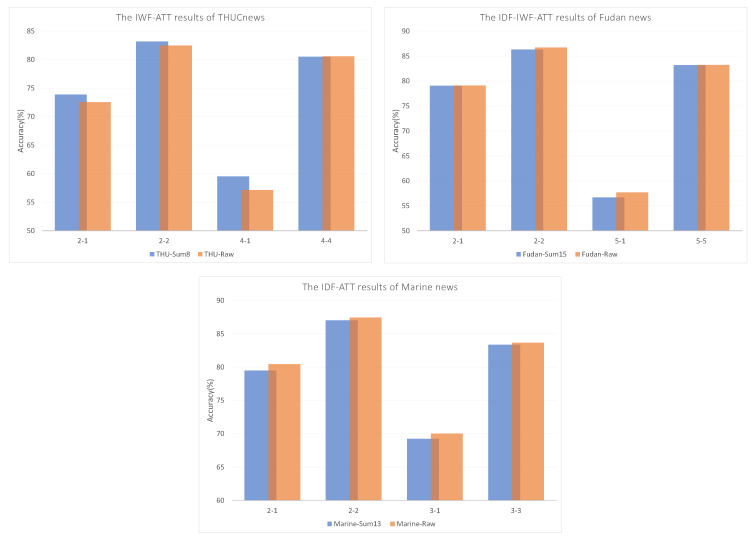
Comparison of results with the optimal solution strategy.

The results under the Sum-H optimal solution strategy were compared to determine the best combination of weights, including IWF-ATT on THUCNews, IDF-IWF-ATT on Fudan news, and IDF-ATT on Marine news. The detailed classification accuracies are displayed in Figure 7. In the 4-way, 1-shot task, the Sum-8 dataset was 2.39% more accurate than the raw text on the THUCNews dataset. The situation deteriorated significantly: raw text was 1% more accurate than the Sum-H dataset only in the 2-way, 1-shot task on the Marine dataset and the 5-way, 1-shot task on the Fudan dataset. The differences in accuracy in the remaining tasks were within 0.5%. Overall, the advantage of the amount of data contained in a single text is insignificant.

### 4.4. Comparison of Computing Time

**Figure 8:**
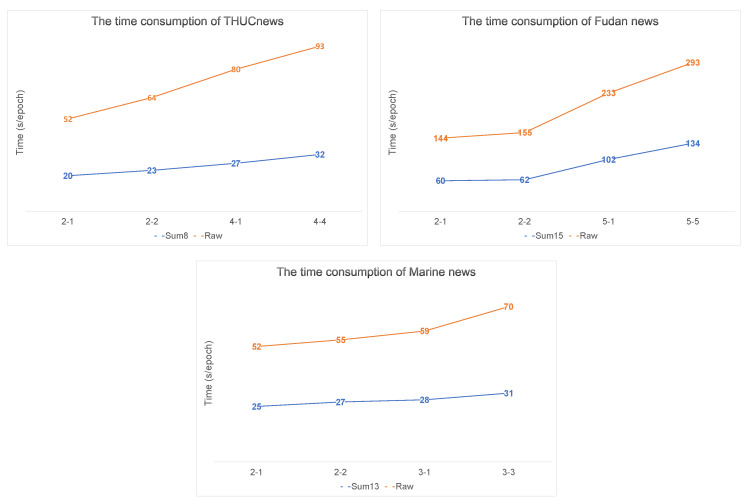
Comparison of time consumption between Raw and Sum-H. These results were estimated using the average time consumption for each epoch.

We illustrate the execution of the proposed model in terms of accelerated training and classification accuracy. The parameters of the input text are listed in Table 5. As a result of the extraction, 32% of the sentences in the Marine dataset were retained as input text. Only 16% of the original text was retained in the other two public datasets. Additionally, the unsupervised sentence extraction method affects reducing text length. The text length of the THUCNews dataset was reduced by 37%, and the text lengths in the Fudan and Marine datasets were reduced by 58%. In addition, the comparison of the training time of each epoch under a specific task is shown in Figure 8. Obviously, Fudan news consumed the longest time. THUCnews was longer than Marine news, but it used less computation time in Sum-H. The training of THUCNews Sum8 data was 60% faster than the training of raw data. Similarly, the speed of the other two datasets was increased by more than 50% using Sum-H text as input.

## 5. Conclusions

We proposed a SumFS model for a few-shot learning task based on unsupervised sentence extraction and feature selection. The unsupervised method reduces the dimensionality of the input text by reducing the amount of irrelevant information, thereby accelerating the model’s training. In the feature selection part, the global feature captures the interaction between categories at the word level, and the local feature mainly captures the contribution of each word to the correct classification. In addition, the ridge regression classifier, which can learn quickly with a few samples, accelerates model training. Under the circumstances of few-shot samples and low dimensional inputs, our framework can achieve stable and even superior performance. When comparing Sum-H and raw text, there was no significant loss of classification accuracy, and raw text conferred no data advantage. Using the Sum-H dataset, our model also showed advantages compared with the baseline models. In addition, it offers a significant improvement in training speed. After dimensionality reduction, the training speed of the model is increased by at least 50%. The experiment results demonstrate the effectiveness of our designed framework and validate our proposition in terms of accuracy and computation speed.

In the future, we will improve the feature selection process and enhance the embedding layer to make the inter-category boundaries more obvious.

## Figures and Tables

**Table 1:** Dataset division. The training and validation data were utilized for training the SumFS model. The unseen target category was applied to evaluate the model. The category numbers were derived from the order in Table 2.

Dataset	Training Category	Validation Category	Target Category
THUCnews	0, 1, 7, 8, 9, 10	4, 5, 6, 12	2, 3, 11, 13
Fudan news	0, 2, 4, 6, 7, 9, 11, 17	1, 3, 10, 13, 18	5, 8, 12, 14, 15, 16, 19
Marine news	0, 1, 7, 8, 9	4, 5, 10	2, 3, 6

**Table 2:** Dataset categories.

Dataset	Category	Average Number of Sentences
THUCnews	Sports, Entertainment, Home, Lottery, ticket, Estate, Education, Fashion, Politics, Constellation, Game, Society, Science and Technology, Stock, Economics	50.36
Fudan news	Agriculture, Art, Communication, Computer science, Economy, Education, Electronics, Energy, Environment, History, Law, Literature, Pharmacy, Military, Mining industry, Philosophy, Politics, Space, Sports, Transport	92.97
Marine news	Marine Equipment, The Blue Economy, Historical culture, Education, Marine Engineering Universities, Marine Military, Marine communication, Inertnet + Ocean, Biotechnology, Travel, High technology	40.78

**Table 3:** The accuracies of different models on Sum-H.

Method	Marine Sum13		THUCnews Sum8		Fudan Sum15	
	3-1	3-3	4-1	4-4	5-1	5-5
MAML	36.02	39.06	28.01	35.68	21.45	21.14
Proto	62.02	78.03	47.90	64.90	51.64	65.33
R2D2	69.08	**83.39**	59.26	80.50	56.43	83.00
MLADA	69.07	82.44	54.16	76.91	53.08	80.76
Ours	**69.22**	83.35	**59.51**	**80.51**	**56.68**	**83.17**

**Table 4:** The accuracies of different weighting methods on Sum-H.

Dataset	N-K	Weight Generator				
		IDF-ATT	IWF-ATT	IDF-IWF-ATT	TF-IDF	IDF-IWF
THU-Sum8	2-1	73.73	73.85	73.83	**74.27**	73.8
	2-2	82.65	83.17	82.92	**83.29**	82.51
	4-1	59.09	**59.51**	58.96	59.09	58.7
	4-4	80.37	**80.51**	80.27	80.31	79.96
Fudan-Sum15	2-1	78.73	78.35	**79.07**	78.34	78.77
	2-2	86.24	86.21	**86.28**	86.09	86.25
	5-1	56.67	56.42	**56.68**	56.21	56.53
	5-5	83.13	83.06	**83.17**	83.14	83.16
Marine-Sum13	2-1	**79.48**	79.31	79.20	79.2	79.35
	2-2	87.04	87.00	87.03	86.73	**87.18**
	3-1	69.22	**69.44**	69.17	68.81	69.33
	3-3	**83.35**	83.23	83.33	83.16	83.33

**Table 5:** The statistical parameters for input text.

Dataset	The Number of Sentences		The Average Length of Texts	
	Sum-H	Raw	Sum-H	Raw
THUCnews	8	50.36	240	382
Fudan news	15	92.97	390	941
Marine news	13	40.78	151	360

## Data Availability

Not applicable.
